# Effects of Transcranial Direct Current Stimulation on Baseline and Slope of Prefrontal Cortex Hemodynamics During a Spatial Working Memory Task

**DOI:** 10.3389/fnhum.2020.00064

**Published:** 2020-04-09

**Authors:** Ryan McKendrick, Brian Falcone, Melissa Scheldrup, Hasan Ayaz

**Affiliations:** ^1^Northrop Grumman Company, Mission Systems, Falls Church, VA, United States; ^2^Department of Psychology, George Mason University, Fairfax, VA, United States; ^3^School of Biomedical Engineering, Science and Health Systems, Drexel University, Philadelphia, PA, United States; ^4^Department of Psychology, College of Arts and Sciences, Drexel University, Philadelphia, PA, United States; ^5^Department of Family and Community Health, University of Pennsylvania, Philadelphia, PA, United States; ^6^Center for Injury Research and Prevention, Children's Hospital of Philadelphia, Philadelphia, PA, United States

**Keywords:** fNIRS, tDCS, working memory, neural efficiency, mixed models

## Abstract

**Background:** Transcranial direct current stimulation (tDCS) has been shown to be an inexpensive, safe, and effective way of augmenting a variety of cognitive abilities. Relatively recent advances in neuroimaging technology have provided the ability to measure brain activity concurrently during active brain stimulation rather than after stimulation. The effects on brain activity elicited by tDCS during active tDCS reported by initial studies have been somewhat conflicted and seemingly dependent on whether a behavioral improvement was observed.

**Objective:** The current study set out to address questions regarding behavioral change, within and between-participant designs as well as differentiating the effects on hemodynamic amplitude and baseline during active tDCS stimulation.

**Methods:** We tested the effects of transcranial direct current stimulation (tDCS) on anterior hemodynamics in prefrontal cortex during performance on a spatial memory task. Prefrontal cortex activity was measured with functional near infrared spectroscopy (fNIRS), a wearable and portable neuroimaging technique that utilizes near infrared light to measure cortical oxygenated and deoxygenated hemoglobin changes non-invasively. There were two groups, one group (*n* = 10) received only sham stimulation and the other group (*n* = 11) received sham followed by anodal stimulation to right ventral lateral prefrontal cortex.

**Results:** Analyses revealed an increase in spatial memory performance following tDCS stimulation. This augmented performance was accompanied by changes to oxygenation (HbO–HbR) at the onset of the hemodynamic response in bilateral dorsolateral prefrontal cortex and left ventral medial prefrontal cortex. In these regions we also observed that stimulation improved neural processing efficiency, by reducing oxygenation and increasing performance from block to block. During and following tDCS stimulation, it was also observed that in bilateral dorsolateral prefrontal cortex the relationship between performance and oxygenation inverted, from a negative relationship to a positive relationship.

**Conclusion:** The results suggest that tDCS is predominately a mechanism for changing neurons propensity for activity as opposed to their strength of activity. tDCS not only alters the efficiency of task relevant processing, but also the nature in which hemodynamic resources are used during augmented task performance.

## Introduction

Non-invasive brain stimulation (NIBS) refers to a family of techniques that aim to alter brain function without the stimulating device being in or on the brain. Most known NIBS techniques include transcranial magnetic stimulation (TMS), and variants of transcranial electric stimulation (tES). TES techniques prescribe the delivery of a small electrical current be passed through the cortex via an anode and a cathode. This is accomplished with either sponges saturated with a conductive solution, or electrodes immersed in a conductive gel that are fixed to specific locations on the scalp or body. This process can be tuned through the electrodes spatial montage which specifies the locations and size of the anodes and cathodes (Wagner et al., 2007; Im et al., 2008) as well as via the strength of the current and the oscillatory nature of the current (i.e., DC, AC, or random) (Paulus, 2011).

There is a growing body of evidence that supports NIBS, and particularly tES as an inexpensive, safe (when administered by professionals) and effective way of augmenting cognition and ameliorating neuropsychological disorders. When transcranial direct current stimulation (tDCS) is applied to the scalp over a targeted brain region it affects the cortical activity and hence the relevant cognitive function associated with that region. The lateral prefrontal cortex is heavily involved in executive function and has been a brain region of interest in many tES studies. Anodal stimulation of the dorsolateral prefrontal cortex has been shown to improve sustained attention (Nelson et al., 2014a,b) and multitasking (Nelson et al., 2016), and anodal stimulation of the ventrolateral prefrontal cortex has been shown to improve visual perceptual learning (Clark et al., 2012; Falcone et al., 2012). In clinical populations tDCS applied to left dorsolateral prefrontal cortex improves scores on the Hamilton Depression Rating Scale, suggesting a reduction in symptoms relating to major depression (Boggio et al., 2008).

Consistent behavioral augmentation following tDCS stimulation has prompted additional neuroimaging research to explain why the technique is effective. Essentially, every imaging modality has been used in conjunction with tDCS to uncover answers to these questions. These include electroencephalogram (EEG), functional resonance magnetic imaging (fMRI), functional near infrared spectroscopy (fNIRS), positron emission tomography (PET), and Magnetoencephalography (MEG). However, despite the relatively consistent behavioral augmentation following tDCS, there has been far less success in finding a consistent pattern of human brain activity in relation to this cognitive enhancement.

The cause of the inconsistencies could be due to the large array of measurement techniques and protocols that can be used. Researchers can examine the effects during both active tasks (Stagg et al., 2009) (i.e., a task involving goal directed behavior), or during rest, where only spontaneous changes in brain activity are monitored (Kwon et al., 2008). For both active and passive task protocols, stimulation has been applied either prior to Antal et al. (2012) or during performance of the task under study (Wirth et al., 2011). The method of applying tDCS prior to a task is predicated on the assumption that the effects of tDCS on cortical excitability extend beyond cessation of stimulation (Nitsche and Paulus, 2001; Snowball et al., 2013). These effects are referred to as “offline-effects” or “after-effects.” Similarly, neuroimaging may occur either during the administration of stimulation (Heimrath et al., 2012), or following the cessation of stimulation (Keeser et al., 2011a,b). Researchers must also consider whether the comparison will be between participants (Meinzer et al., 2012), within participants (Holland et al., 2011), or both (McKendrick et al., 2015).

Due to the aforementioned sources of potential variability the neurophysiological effects of tDCS paired with a cognitive task are inconsistent. For instance, on a change detection task anodal tDCS can reduce alpha and gamma power (Marshall et al., 2016). In another study, tDCS paired with an n-back memory task reduced prefrontal oxygenated (HbO) and deoxygenated hemoglobin (HbR) (Choe et al., 2016). Yet, there is also evidence of increased alpha power (Choe et al., 2016), and BOLD response with anodal stimulation (Alekseichuk et al., 2016; Hauser et al., 2016).

Importantly, all these previously reported effects occurred in the absence of objective behavioral enhancement which may account for some of the variability in the results of these studies and certainly begs the question as to whether these neurophysiological changes are reflective of tDCS-induced augmented cognition. When behavioral enhancement has been observed, tDCS more often than not, reduces brain activity and EEG alpha power. For example, during a memory match to sample task increased performance co-occurred with reduced alpha power (Heimrath et al., 2012). During picture naming tasks it's been observed that anodal stimulation can increase reaction time and this was associated with reduced BOLD response in left inferior frontal sulcus (IFS) (Holland et al., 2011), and increased duration of IFS activity (Holland et al., 2016). Additionally, during a spatial memory task, a recovery of task performance was associated with decreased oxygenation in right dorsomedial and dorsolateral PFC (McKendrick et al., 2015). Finally, during a go no-go inhibition task cathodal stimulation was found to increase reaction time and increase activity in right M1 (Garcia-Cossio et al., 2016). The negative relationship between task performance and brain activity in the region targeted by anodal stimulation is inconsistent with many tDCS studies that did not observe behavioral change and suggests an effect associated with enhanced efficiency rather than increased neural activation.

In addition to behavioral change, differential effects on behavior and components of the hemodynamic response due to within or between-participant designs have not been fully explored. The majority of concurrent behavioral and neurophysiological changes have observed these effects only within-participants. Yet, the bulk of behavioral enhancement has been observed in between-participants paradigms where no neuroimaging methods were applied. One study, testing word generation did observe a negative relationship between number of words generated and targeted brain activity between participants (Meinzer et al., 2012), but the design did not allow for comparisons of between and within participants designs. Other studies have employed mixed participant designs but have not contrasted the effects associated with each design (McKendrick et al., 2015).

In this study we applied tDCS over the ventral PFC to replicate the behavioral benefits observed in previous studies. We used concurrent functional infrared spectroscopy (fNIRS) to observe brain activity under this enhanced condition. Additionally, we examined these effects within and between participants. This was done to reconcile the different techniques between previous behavioral and neuroimaging studies of tDCS augmentation. We hypothesized that in a mixed design, tDCS will increase performance within the stimulation group overtime, as well as between the stimulation and sham groups at specific time points. We also analyzed whether behavioral augmentation increases overall resource availability (hemodynamic baseline), or the strength of brain activity (hemodynamic amplitude). Previous research has shown that tDCS applied at the cellular level predominantly effects resting membrane potentials by modulating the conductance of sodium and calcium channels (Nitsche et al., 2003; Bikson et al., 2004; Stagg and Nitsche, 2011). Given the previously referenced cellular and cognitive neuroscience findings we hypothesized that if tDCS stimulation resulted in enhanced spatial working memory performance then we would observe an associated decrease in activation reflecting enhanced neural efficiency.

## Methods

### Participants

Twenty-one George Mason University Students (10 males and 11 females) aged 18–35 years (mean = 20.3 years) were recruited. Participants were randomly placed in either an active or sham stimulation group. Eleven participants were placed in the active stimulation group and 10 participants were placed in the sham stimulation group. Admittedly, this is a modest sample size for testing between group effects and has less of an effect on our within group analyses. Furthermore, this sample size is on the higher end of the median (*n* = 18) reported for similar studies in a meta-analysis (Hill et al., 2016). All participants reported being right handed with normal or corrected-to-normal vision. All participants reported not taking prescriptions drugs which alter neural or cognitive function. This research complied with the American Psychological Association Code of Ethics. All participants provided informed consent prior to participation. The study was approved by George Mason University's Institutional Review board.

### Spatial Memory Task

**Figure 1:**
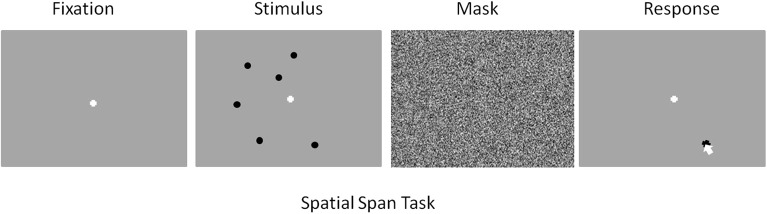
Screenshot examples for each part of the spatial memory task in order of presentation. From left to right: Fixation (15 s), Stimulus (1 s), Mask (4 s), Response (space bar press).

The stimuli for the spatial task consisted of black circles presented simultaneously over a gray background. Figure 1 depicts a typical trial from this task. The number of circles presented varied across trials. All participants began with spatial span load being randomly assigned as five, six, or seven circles for a given trial. The spatial location for each circle was randomly chosen with the only caveat being that the center of one circle had to be >150 pixels from the center of any other circle displayed. The start of each trial began with a white fixation cross presented over a gray background this display was maintained for 15 s. The length of fixation was chosen to allow ample time for a participant's hemodynamic response to return to baseline after responding to a previous stimulus. This was followed by presentation of the spatial stimulus which persisted for 1 s. All circles were presented simultaneously across the screen. This was followed by a random noise mask which was sustained for 4 s., after which a gray screen was presented and participants used a mouse to recall the locations of the circles by clicking with as much precision as possible where they remembered seeing the circles (Figure 1). Once the participant believes they have answered completely, they press the space bar to move onto the next trial. This task consisted of a total of 109 trials broken down into 10 practice trials and three experimental blocks which consisted of 33 trials each.

Performance on the spatial span task was calculated based on the center to center distance of a recalled location and a presented circle. A recalled location was scored as a correct response if its center was within 300 pixels of a presented circles center. From this data two measures of spatial span performance were calculated. The first measure, recall probability was calculated as the number of recall responses for a given trial that were scored as correct (per the aforementioned criteria) divided by the number of circles presented. The second measure, spatial error was calculated as the center to center distance (in pixels) between a presented circle and the nearest recall response divided by the number of circles presented.

### Procedures

**Figure 2:**
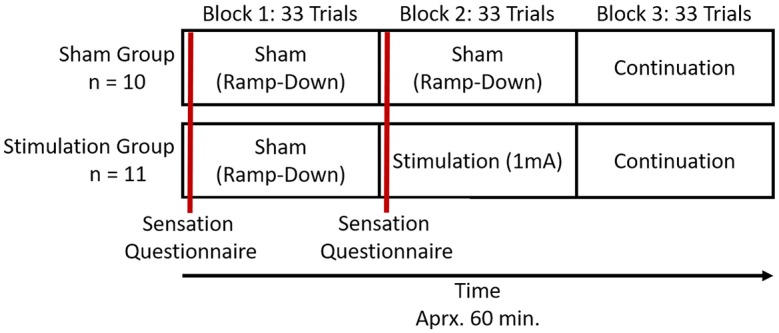
Representation of trial and stimulation structure for the study.

A graphical representation of the experimental procedure can be found in Figure 2. Participants were randomly assigned to two experimental groups, sham and stimulation. Participants in both groups were only told they would receive a dosage of electrical current during the course of the study. Participants first performed the 10 trials of practice where no imaging or stimulation occurred. They then completed the three experimental blocks of 33 trials in succession. There was ~60 s between blocks as the experiment initiated the next experimental block. Participants were imaged with fNIRS in all three experimental blocks. In the first experimental block both the stimulation and sham groups received sham tDCS. In the second experimental block the sham group once again received sham tDCS, but the stimulation group received active stimulation of 1 mA. In the third experimental block both groups received no tDCS and were monitored for continuation effects (i.e., after-effects). None of the imaging or stimulation equipment was removed for the third experimental block. The total experiment took ~60 min. We chose to use these within group procedures to provide clear points of comparison between the two groups (i.e., blocks 1 and 3 are identical), and maximize blinding effects. By only providing active stimulation in the second block both groups will have perceived sham stimulation equivalently. Only the stimulation group during their second block could have seen through the blinding, but by that time they will have already performed their sham block.

### tDCS Montage

**Figure 3:**
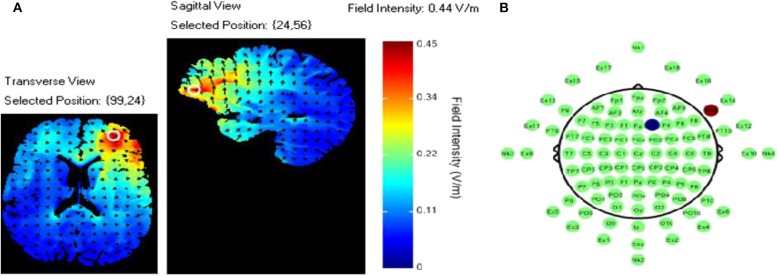
**(A)** Current flow model. **(B)** TDCS montage: the red dot represents the anodal electrode over the F10 and the blue dot represents the cathodal electrode over the F2.

TDCS was applied using an ActivaDose II Iontophoresis Delivery Unit (ActivaTek Inc. Salt Lake City, UT), which provides for delivery of a constant low level of direct current. Participants were fitted with an elastomere cap with high density tDCS (HD-tDCS) electrode holders (Soterix Medical Inc., New York, NY; www.soterixmedical.com). Ag/AgCl sintered ring electrodes were placed in each holder along with electroconductive gel to conduct the current to the scalp (see Villamar et al., 2013 for a more detailed description of the Soterix HD-tDCS system). A pilot study monitoring this task with fNIRS identified a region of right VLPFC that showed a significant relationship between this brain region and task performance (McKendrick et al., 2015). Using the modeling software HD- explore (Soterix Medical Inc., New York, NY), we constructed a montage that elicited maximum current flow to right VLPFC by placing the anode at F10 and cathode at F2 in the 10-20 EEG system (McKendrick et al., 2015). A visualization of the current flow model and the subsequent montage that was used can be seen in Figure 3, respectively. Active stimulation applied 1 mA to the anode for 15 min. One mA was used due the high current density of the montage (0.44 V/m over VLPFC). During initial testing we found higher current doses to be intolerable to participants. Sham stimulation consisted of an automatic ramp-up to 1 mA (this occurred over ~30 s), which was immediately ramped down to zero current (this also occurred over ~30 s) and the stimulation delivery unit was turned off. The delivery unit and readings regarding its parameters were hidden from study participants to aid in blinding them to their stimulation/sham group membership.

### Sensation Questionnaire

A sensation questionnaire was administered at two different time points throughout the tDCS application. The first was administered 1 min after the onset of the stimulation in the first experimental block, and the second, 1 min after the onset of stimulation in the second experimental block. Only two surveys were administered as the third block of the study did not contain any active or sham stimulation and was used to assess the after effects of active or sham stimulation applied during block 2. Participants were asked to rate their perceived sensations of itching, heat/burning, and tingling on a 10-point rating scale; a response of 1 indicated that no sensation was experienced and 10 indicating extreme sensation. Stimulation was to be stopped immediately if participants reported a seven or above on any of the sensation measures (This did not occur with any of the participants) (Falcone et al., 2012).

### fNIRS

**Figure 4:**
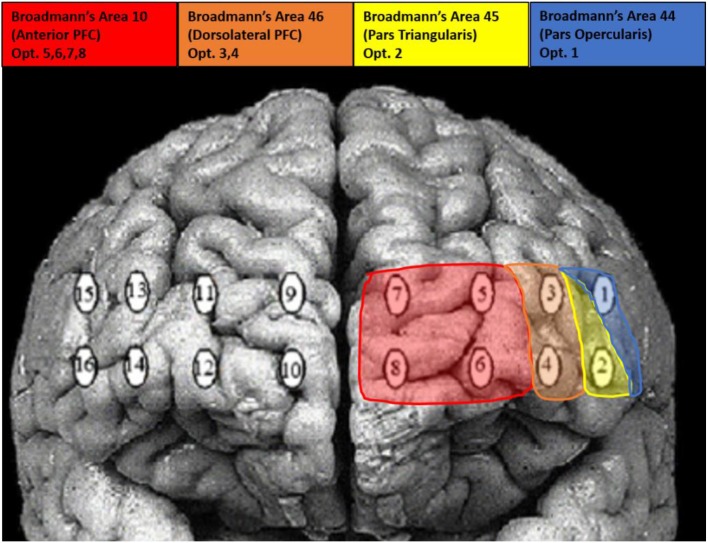
Visualization of optode locations and Broadmann's Area's.

Raw light intensities were acquired through an fNIR Devices fNIR 1000 system (fNIR Devices LLC, Photomac MD; https://fnirdevices.com/) composed of four light emitters and eight photodetectors. This configuration yields 16 dual-wavelength optodes emitting near infrared wavelengths of 730 nm and 850 nm. The imaging temporal resolution was 2 Hz and the average emitter to detector separation distance was 2.5 cm allowing for light penetration of ~1.25 cm deep into the human head. Concurrent imaging with our tDCS setup required the imaging device be placed over the elastomere cap covering the scalp to provide imaging of frontal cortical regions (Figure 4). The cap did not interfere with imaging and at worst may have reduced the light penetration of our device by ~1 mm. COBI Studio software (Drexel University) was used for data acquisition and visualization of data quality during imaging (Ayaz et al., 2011).

Each participant's raw light intensities were low-pass filtered with a finite impulse response, linear phase filter with order 20 and cut-off frequency of 0.1 Hz to attenuate high frequency noise associated with respiration and cardiac cycle effects (Ayaz et al., 2010). The data was checked for any potential optode saturation (when light intensity at the detector was higher than the analog-to-digital converter limit) and motion artifact contamination by means of a coefficient of variation based assessment (Ayaz et al., 2010). Relative chromophore concentrations were calculated by submitting the filtered light intensities to the modified Beer-Lambert law (Ayaz et al., 2012) to estimate total photon path length of the different wavelengths of back-scattering light.

The group average temporal window of the hemodynamic response was determined for use in later statistical analyses by averaging trial time series across participants. Visual inspection of the average trial time series revealed that the peak positive concentrations of HbO and negative concentrations of HbR were observed between seven and 12 s post stimulus presentation and this time span was selected as our temporal window. We wanted to represent both the onset and peak of the hemodynamic response to assess tDCS mechanisms hypothesized to alter baseline hemodynamics (i.e., chromophore concentrations at the onset of the response) and/or the magnitude of the hemodynamic response (i.e., chromophore concentrations at the peak of the response).

### Data Analysis

#### Linear Mixed Effects Models

All forthcoming statistical tests employ linear mixed effects models implemented in R (R Core Team, 2012) via lme4 (Bates et al., 2014). Linear mixed effects estimates were computed with restricted maximum likelihood. Denominator degrees of freedom and *p*-values were estimated via Sattherwaite corrections implemented via lmerTest (Kuznetsova et al., 2013).

#### Fixed and Random Effects Selection

Bayesian information criterion (BIC) was used to select the fixed and random effects in the final models for each dependent variable. Competing models were constructed by adding potentially meaningful random and fixed effects to a null model. The null model was specified in each case as having no fixed effects and a random effect of participant intercept. All competing models were estimated with maximum likelihood to allow for testing of fixed effects. The competing models were tested simultaneously with BIC and the strength of evidence criterion described by Kass and Raftery (1995) was employed. In the procedure deviations of >2 BIC are viewed as a meaningful difference. The final model was selected based on having the lowest BIC, with no other models of interest having a BIC deviance of <2 (McKendrick et al., 2017).

#### Multiple Comparisons Corrections

In all forthcoming fNIRS analyses multiple comparisons were corrected for across hypotheses and optodes but within chromophores by adjusting *p*-value criterion with false discovery rate (FDR) corrections. The Benjamini-Hockberg FDR procedure was employed with alpha set to 0.05 in the equation.

#### Behavioral-Hemodynamic Interaction

Our hypotheses dictated how we analyzed the relationships between behavioral performance, the oxygenation concentration at the onset of the hemodynamic response, and the change in oxygenation concentration with the hemodynamic response. Analyses of the interactions between hemodynamic and behavioral observations required down-sampling of the higher frequency hemodynamic signal. Therefore, we performed trial by trial regression for each participant to calculate oxygenation intercepts and slopes for the hemodynamic temporal window. These intercepts and slopes were used as outcome variables in all forthcoming behavioral-hemodynamic interaction analyses. The intercepts were taken to represent baseline hemodynamic resources, and the slopes were taken to represent the magnitude of a hemodynamic response.

## Results

### Behavioral

#### Recall Probability

The most parsimonious linear mixed effects model as tested by the BIC strength of evidence criteria specified a linear effect of experimental block, a main effect of stimulation group and interaction between block and stimulation group. Random effects of participant and memory load (i.e., trial difficulty) were also selected. Planned regression contrasts are dummy coded with the stimulation group as the comparison condition so we can reject the null-hypothesis of no change in slope for the stimulation group.

**Figure 5:**
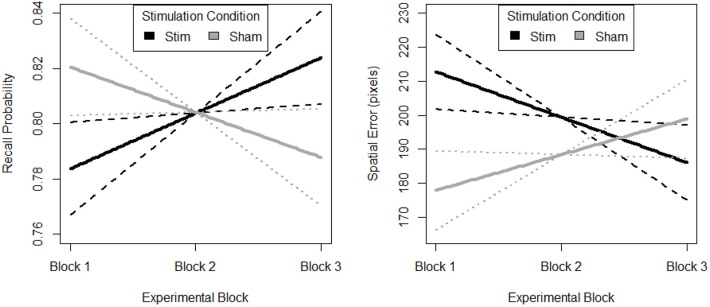
Behavioral performance (Left. probability, Right. error) on spatial memory task. Block 1, both groups receive sham, block 2, stim group received stimulation, sham group received sham, and block 3, both groups were monitored for 33 additional trials of the task. Solid lines depict fixed effects regression slopes, dashed lines represent 95% confidence bands of regression estimate.

The random effect of participant suggests that individuals differed in their initial task performance. Further, initial performance was also influenced by the memory load of the trial. However, after accounting for this random variance there was still a parsimonious fixed effect of block and stimulation group. The fixed effects estimates are visualized in Figure 5. The stimulation group intercept was greater than zero (*df* = 8.26, *B* = 0.76, *SE* = 0.05, *p* < 0.001). Estimates of the sham groups ability were not significantly different (*df* = 30.05, *B* = 0.07, *SE* = 0.05, *p* > 0.05). The stimulation group increased their recall performance throughout the experiment, as evidenced by a significant positive linear slope (*df* = 2054.06, *B* = 0.02, *SE* = 0.01, *p* < 0.001*)*. Further, this increase was significantly greater than that seen in the sham group (*df* = 2054.02, *B* = −0.04, *SE* = 0.01, *p* < 0.01*)*.

#### Spatial Error

The most parsimonious linear mixed effects model as tested by the BIC strength of evidence criteria specified a linear effect of experimental block, a main effect of stimulation group and interaction between block and stimulation group. Random effects of participant and memory load (i.e., trial difficulty) were also selected. Planned regression contrasts are dummy coded with the stimulation group as the comparison condition so we can reject the null-hypothesis of no change in slope for the stimulation group.

The random effect or participant suggests that individuals differed in their initial task performance. Further, initial performance was also influenced by the memory load of the trial. However, after accounting for this random variance there was still a parsimonious fixed effect of block and stimulation group. The fixed effects estimates are visualized in Figure 5. The stimulation group intercept was greater than zero (*df* = 7.97, *B* = 226.10, *SE* = 35.57, *p* < 0.001). Estimates of the sham groups ability were not significantly different (*df* = 30.34, *B* = −58.65, *SE* = 35.65, *p* > 0.05). The stimulation group decreased their spatial error throughout the experiment, as evidenced by a significant negative linear slope (*df* = 2054.07, *B* = −13.33, *SE* = 5.90, *p* < 0.05*)*. Further, this decrease was significantly greater than that seen in the sham group (*df* = 2054.03, *B* = 23.82, *SE* = 8.15, *p* < 0.01*)*. Also, there is no evidence of a difference in initial performance between the two groups.

### Hemodynamics

To highlight the hemodynamic effects of the stimulation group's second experimental block we included a variable in the mixed model selection procedure that aggregated experimental block and stimulation group into a six-level factor. Here after we refer to this variable as the conditional aggregate, and future analyses with this variable use dummy coded contrasts with the comparison condition represented by the stimulation group's second experimental block. We observed that the effects across HbO and HbR were negatively correlated, which is as expected assuming they were associated with brain activity. Therefore, we calculated changes in oxygenation (i.e., the difference between HBO and HbR) as an additional dependent variable.

**Figure 6:**
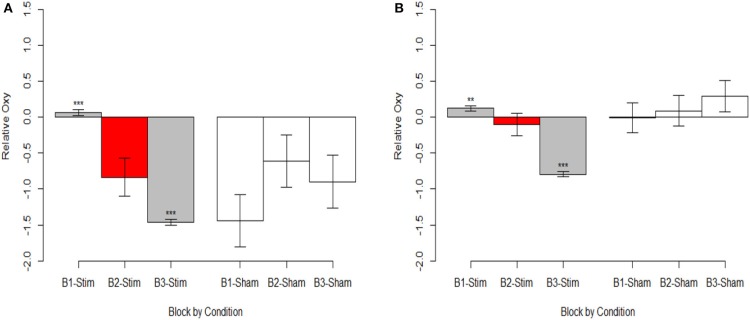
Oxygenation at onset of hemodynamic response. **(A)** Optode 1 left dorsolateral BA 44/45. **(B)** Optode 15 right dorsolateral BA 44/45. Red bar represents comparison condition for dummy coding in mixed effects regression models. **p < 0.01, ***p < 0.001.

**Figure 7:**
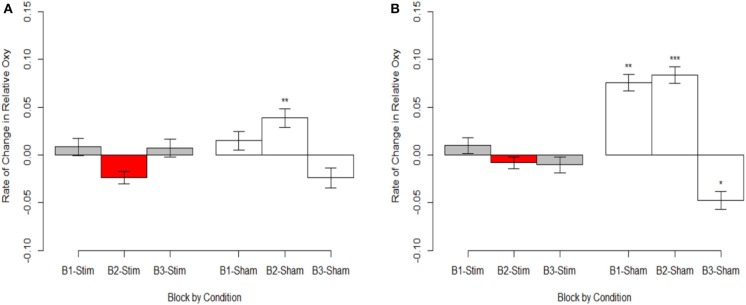
Magnitude of oxygenation change during hemodynamic response. **(A)** Optode 1 left dorsolateral BA 44/45. **(B)** Optode 15 right dorsolateral BA 44/45. Red bar represents comparison condition for dummy coding in mixed effects regression models. *p < 0.05, **p < 0.01, ***p < 0.001.

The most parsimonious linear mixed effects models as tested by the BIC strength of evidence criteria specified a full factorial interaction between the conditional aggregate and within trial fNIRS samples (i.e., the timeseries of fNIRS samples for the hemodynamic window we defined as 7 to 12 sec. after stimulus presentation). Random effects of participant and trial slope uncorrelated with participant intercept were also selected. This model was selected as most parsimonious for all three dependent variables, HBO, HbR, and Oxygenation. The results of the fixed effects analyses are reported in Supplementary Table 1 and visualized in Figure 6 (intercepts) and Figure 7 (slopes).

### Behavioral-Hemodynamic Interaction

To highlight the effects of the stimulation group's second experimental block we mean centered our variable for time (i.e., block). Therefore, intercepts reported for these analyses reflect differences during the second experimental block.

**Figure 8:**
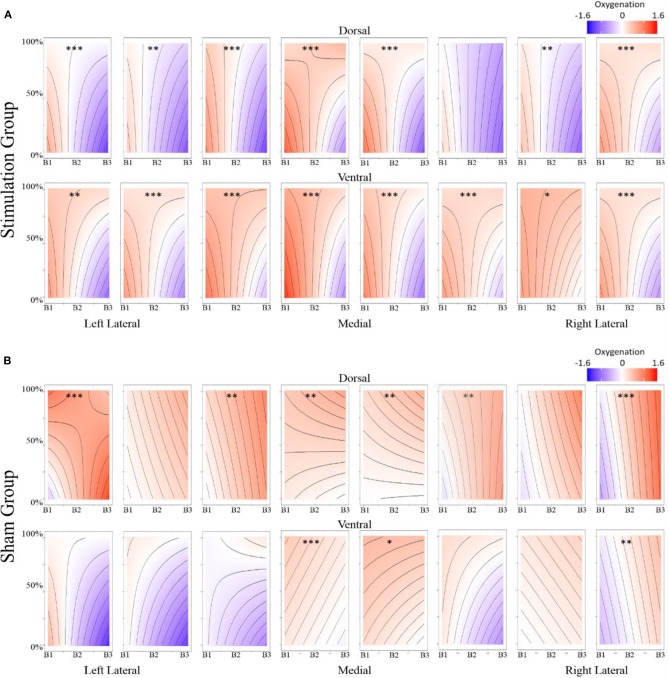
**(A)** Stimulation condition three-way interaction between relationship of spatial memory accuracy and oxygenation as a function of experimental block. Dorsal optodes are odd numbered, ventral optodes are even numbered. Optodes are ordered from least on the left to greatest on the right. *p < 0.05, **p < 0.01, ***p < 0.001. **(B)** Sham condition three-way interaction between relationship of spatial memory accuracy and oxygenation as a function of experimental block. Contrast between sham and stimulation conditions, *p < 0.05, **p < 0.01, ***p < 0.001.

**Figure 9:**
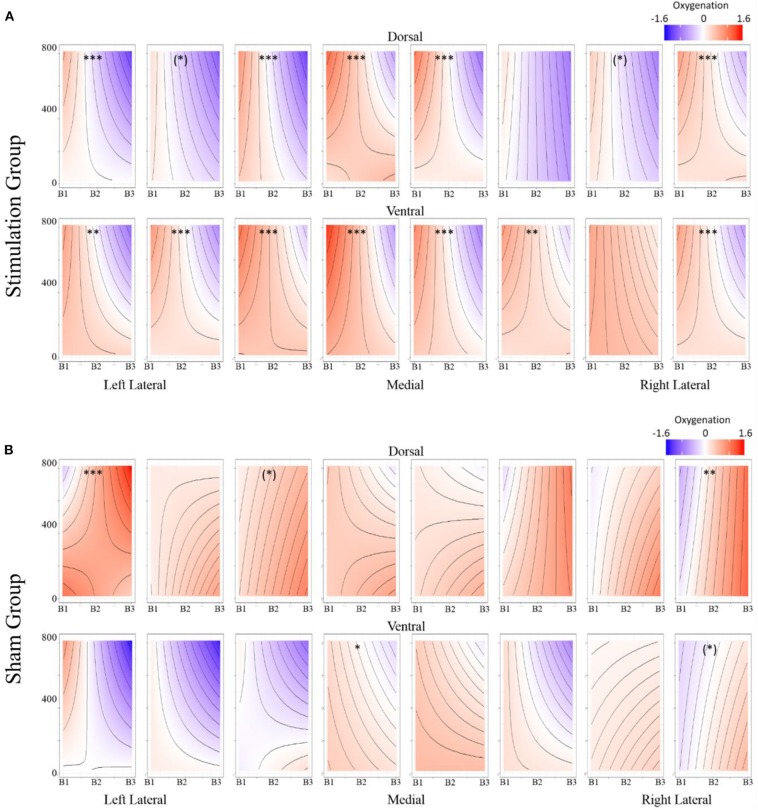
**(A)** Stimulation condition three-way interaction between relationship of spatial memory error distance and oxygenation as a function of experimental block. Dorsal optodes are odd numbered, ventral optodes are even numbered. Optodes are ordered from least on the left to greatest on the right. *p < 0.05, **p < 0.01, ***p < 0.001. **(B)** Sham condition three-way interaction between relationship of spatial memory error distance and oxygenation as a function of experimental block. Contrast between sham and stimulation conditions, *p < 0.05, **p < 0.01, ***p < 0.001.

The most parsimonious linear mixed effects models as tested by the BIC strength of evidence criteria specified a full factorial interaction (performance measure, experimental group, and experimental block) for hemodynamic onset. The random effect of participant intercept was also selected. For the hemodynamic slope the most parsimonious model was the null model for both behavioral measures specifying only a random effect of participant intercept and will not be analyzed further. The fixed effects estimates for models are presented in Supplementary Table 2, the results are visualized in Figure 8 for recall probability, and Figure 9 for spatial error.

## Discussion

Non-invasive brain stimulation (NIBS) aims to alter brain function to enhance human behavior in work and clinical environments. Evidence supports NIBS implemented via transcranial direct current stimulation (tDCS) is an inexpensive, safe and effective way of achieving this goal. However, tDCS requires foresight regarding functional brain mapping, current flow, experimental design, and evidence of behavioral augmentation. Our study addressed behavioral and hemodynamic changes caused by tDCS stimulation. We also addressed the influence of within and between-participant designs. Furthermore, we documented effects on oxygenation concentration at the onset of the hemodynamic response, and the magnitude of change in oxygenation during the hemodynamic response.

### Behavioral Effects

We observed that simulation of right ventrolateral PFC (VLPFC) had positive effects on both how accurately and how precisely participants were able to encode, maintain, and recall spatial locations. In a similar study, using the same task (but with different condition orders) and equivalent stimulation parameters, performance initially declines and tDCS stimulation only aided in the recovery of performance (McKendrick et al., 2015). In light of this, the current study needed to provide evidence of stimulation enhancing performance both within and between participants. This was observed for both accuracy and precision. Participants in the stimulation group demonstrated improvement relative to their own baseline, and relative to the rate of change observed in the sham group. This supports our hypothesis for positive behavioral augmentation following tDCS stimulation to right ventrolateral prefrontal cortex (VLPFC).

Evidence of behavioral change is important for two reasons. First, enhanced accuracy and precision with stimulation meets our necessary condition of observing behavioral enhancement. This afforded further analysis of the hemodynamic effects of tDCS. Second, it adds to previous findings that focused tDCS stimulation to right VLPFC enhances aspects of spatial working memory (McKendrick et al., 2015). Specifically, we observed a 7.8% increase in accuracy and a 21.5% increase in precision for stimulation within participants (these values are derived from the intercept and slope estimates of the main effects in the behavioral linear mixed effects models by converting the log-odds ratios to probabilities). The benefit of stimulation may be even greater when considering the between participants effects. Specifically, noting the decrement experienced by those in the sham group during prolonged task performance. If stimulation had been performed on a task that doesn't have fatigue/vigilance effects, the benefits to accuracy may be as high as 15.7 and 46.2% for precision (these values are derived from the intercept and slope estimates of the interaction effects in the behavioral linear mixed effects models). These are meaningful improvements. In the span of 1 h, they approach improvements previously observed after 10 h of practice (without stimulation) on a very similar spatial memory task (McKendrick and Parasuraman, 2014).

### Hemodynamic Effects

We observed inverse relationships between HbO and HBR, and directionally consistent effects in Oxy provide strong evidence that our observed effects were associated with brain activity. This concurrence of effects is a stronger representation of operationalized brain activity because the bold response is defined by concurrent effects in HbO and HbR. These effects are preferable to the common practice of only reporting HbO. While HbO is correlated with the BOLD response (Huppert et al., 2006; Steinbrink et al., 2006), HbO is also heavily effected by motion and physiological artifacts (Kirilina et al., 2012).

Stimulation had consistent effects across HbO, HbR, and Oxy which were consistent with tDCS imaging studies that also report behavioral augmentation. Reductions in PFC oxygenation followed two trends that were slightly different. Each trend depended on the comparisons made and represents different aspects of neurological activity. Essentially, we observed different effects depending on whether the effects were between-participants, or within-participants. When exploring effects between individuals, we observed that during the time period of stimulation the magnitude of the hemodynamic response was suppressed for those undergoing stimulation (see Figure 7). This effect was not observed within stimulation participants. However, within stimulation participants we did observe reduced hemodynamic resources at the start of the maintenance period of the memory task. This effect increased in magnitude throughout the course of the study (see Figure 6). These decreases in brain activity in the stimulation group are consistent with a number of studies also observing behavioral effects (Holland et al., 2011; Heimrath et al., 2012; McKendrick et al., 2015). They also support our initial hypothesis of increased neural efficiency following tDCS induced performance enhancement. However, to further interpret these effects we wanted to explore them in direct relation to the changes we observed in performance between the stimulation and sham groups.

### Behavior-Hemodynamic Interactions

Overall, our hemodynamic results cohere with the majority of tDCS imaging studies of similar design, and behavioral outcomes. When tDCS is administered concurrently with imaging and task performance, and performance increases; brain activity decreases. In two instances faster picture naming has been associated with reduced activity in inferior frontal sulcus following anodal tDCS applied to FC5 (Holland et al., 2011, 2016). Similarly for word generation, reduced activity followed anodal tDCS applied to inferior frontal gyrus (Meinzer et al., 2012). Deactivation was also observed in right DLPFC following anodal tDCS to right VLPFC and increased spatial memory performance (McKendrick et al., 2015).

However, these instances of deactivation are at odds with similar studies that did not find changes in behavior. In a number of visual tasks that applied anodal tDCS to either occipital, parietal or motor cortex, increased brain activity was observed in these brain regions (Alekseichuk et al., 2016; Callan et al., 2016; Lindenberg et al., 2016; Falcone et al., 2018). This was similar for an n-back and flight simulation task where prefrontal activity either increased or decreased depending on where anodal or cathodal tDCS was applied (Choe et al., 2016). Yet in each of these instances neurological changes were not accompanied by a significant behavioral improvement between stimulation groups.

**Figure 10:**
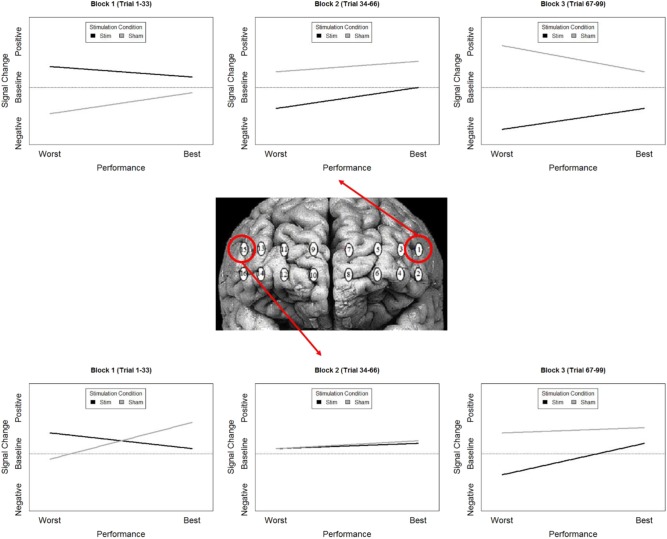
Simplified representation of changes in the relationship between Oxy signal change and performance in Optodes 1 and 15.

While our effects generally suggest anodal tDCS induces deactivation, interactions with performance and current dose suggest a more nuanced interpretation. When testing the effects of performance on the modulatory effects of tDCS we initially observed a negative relationship between performance and oxygenation which became a positive relationship during and after exposure to tDCS (Figure 10). This effect was strongest in bilateral Pars Opercularis (optodes 1 and 15), this is particularly interesting considering right pars Opercularis is very close to the maximal current density predicted by our current modeling for the administered stimulation montage. While both brain regions reduced the amount of oxygenation at the onset of the hemodynamic response, and the relationship between oxygenation and performance inverted, optimal performance was achieved with a slight increase in oxygenation for right pars Opercularis, and decrease in oxygenation for left pars Opercularis. It's possible that this difference was due to left pars Opercularis being contra lateral to the region of maximal current density.

To further understand the effects of tDCS, we wanted to look at more than overall oxygenation, but also the components of the hemodynamic response that are most effected by tDCS. The prime candidate components of this would be the magnitude of the hemodynamic response and the amount of oxygenation present prior to the hemodynamic response. In our analyses we only observed significant changes in the amount of oxygenation present prior to the hemodynamic response. Specifically, when accounting for effects of time and performance only changes at the onset of the hemodynamic response differed between stimulation and sham groups. This is consistent with the categorization of tDCS stimulation as a mechanism for priming brain activity for enhanced processing. *In vitro* testing of tDCS also supports this. Observations of increased resting membrane potentials via anodal stimulation have been related to local reductions in GABA neurotransmitters, where decreases in resting potentials via cathodal stimulations are associated with reduced activity at synapses that respond to glutamate (Stagg et al., 2009). The continued effects of anodal tDCS post current cessation are also dependent on modulation of both GABAergic and glutamatergic synapses, specifically for synapses on interneurons within the cortex (Stagg and Nitsche, 2011).

**Figure 11:**
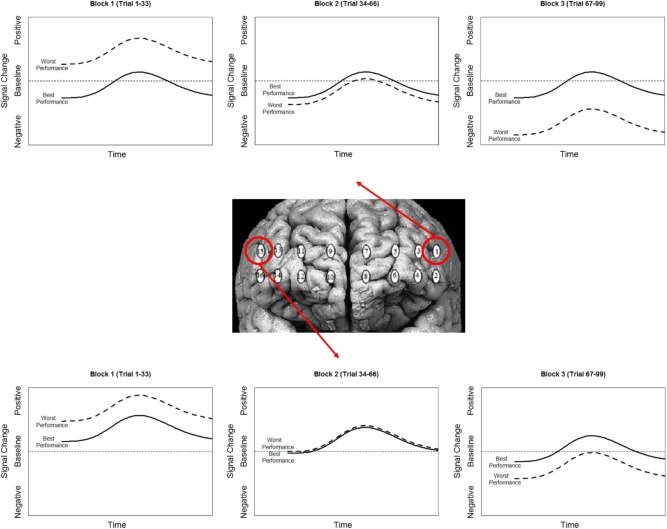
Simplified representation of the effect of stimulation (contrast between stim and sham groups) on the hemodynamic response and its association with performance in optodes 1 and 15.

Combining these observations affords a more precise description of the neuro-cognitive outcomes for short-term performance enhancement following tDCS. Specifically, via a priming effect, tDCS appears to lower activity in specific brain regions, effectively increasing their processing efficiency. Concurrent with this efficiency increase, there is also appears to be a threshold shift. This could be thought of as raising the relative ceiling on processing allowing greater (relative) activity to increase performance (Figure 11).

### Limitations

We employed a prior neuroimaging and current modeling to direct current to regions of the brain associated with performance on the spatial memory deployed in this study. This was done in light of evidence showing that placement of the anode or cathode over a region of interest during tDCS produces current flow models with near-global changes in cortical membrane potentials (Datta et al., 2009). In spite of our attempt to localize the effects of stimulation, bilateral effects were observed. Based on current flow models of our montage (anode at f10, cathode at f2) the current was anticipated to be localized to right VLPFC, with some current also flowing to right ventromedial PFC. However, our current model (Figure 2) did show a non-zero field intensity in left PFC. While we did not explicitly test whether current reached left lateral PFC, we did observe hemodynamic effects in this region. The presence of these effects suggests that minimally, this current was sufficient to effect brain activity across PFC. It is possible that by using a superior montage that creates a more focal current may yield different results relative to those observed here.

## Conclusion

We set out to induce behavioral augmentation following fNIRS targeted anodal tDCS to right VLPFC and through concurrent neuroimaging describe the effects of stimulation on cerebral hemodynamics. We successfully improved performance on a spatial memory task. The augmented performance was accompanied by changes to oxygenation (HbO-HbR) at the onset of the hemodynamic response in bilateral dorsolateral prefrontal cortex and left ventral medial prefrontal cortex. This suggests that tDCS is predominately a mechanism for changing neurons propensity for activity as opposed to their strength of activity. Further, in these regions we observed that stimulation improved neural processing efficiency, by reducing oxygenation and increasing performance. Finally, tDCS also had a positive relationship between oxygenation and task performance. This provides evidence that tDCS may alter the efficiency of task relevant processing, and also the nature in which hemodynamic resources are used during augmented task performance.

## Data Availability Statement

The datasets generated for this study are available on request to the corresponding author.

## Ethics Statement

This study was carried out in accordance with the recommendations of Declaration of Helsinki, George Mason University Institutional Review Board with written informed consent from all subjects. All subjects gave written informed consent in accordance with the Declaration of Helsinki. The protocol was approved by the George Mason Institutional Review Board.

## Author Contributions

Tasks were designed by RM. Data collection was completed by RM and MS. Data analysis was completed by RM. The final manuscript was prepared by RM, MS, BF, and HA.

### Conflict of Interest

fNIR Devices, LLC manufactures the optical brain imaging instrument and licensed IP and know-how from Drexel University. HA was involved in the technology development and thus offered a minor share in the startup firm fNIR Devices, LLC. RM and BF were employed by Northrop Grumman during the preparation of the manuscript. The authors declare that this study received funding from Northrop Grumman. The study was designed, and data was collected, analyzed and interpreted prior to the authors employment at Northrop Grumman. Northrop Grumman was not involved in the study design, data collection, analysis and interpretation. The remaining author declares that the research was conducted in the absence of any commercial or financial relationships that could be construed as a potential conflict of interest.
